# Adult Medulloblastoma Presenting With Audiovestibular Symptoms and an Alternating Unterberger Test

**DOI:** 10.7759/cureus.94670

**Published:** 2025-10-15

**Authors:** Christopher P Bunting, Trung Nguyen, Elliot Benjamin, Maria Thom, Jeremy Rees

**Affiliations:** 1 Neurosciences Institute, Cleveland Clinic London, London, GBR; 2 Paediatric Oncology, Great Ormond Street Hospital, London, GBR; 3 Otolaryngology, Charing Cross Hospital, London, GBR; 4 Neuropathology, National Hospital for Neurology and Neurosurgery, London, GBR

**Keywords:** adult medulloblastoma, central vertigo, neurology and neuro-oncology, proton beam radiotherapy, vestibular dysfunction

## Abstract

A man in his 30s presented with a four-week history of vertigo, unilateral tinnitus, and nausea on a background of a three-year history of asymmetric audiovestibular symptoms attributed to peripheral vestibular pathology. The neurological examination was normal. A brain magnetic resonance imaging (MRI) demonstrated a fourth ventricular enhancing tumour, which was resected. The final histology was classical medulloblastoma CNS WHO Grade 4. He received craniospinal irradiation and adjuvant chemotherapy. Two years after treatment, he relapsed with spinal drop metastases and received palliative treatment. This case highlights that adults with medulloblastoma may present atypically with a longer history of unusual audiovestibular symptoms rather than signs of raised intracranial pressure or brainstem/cerebellar dysfunction.

## Introduction

Medulloblastoma is a malignant, embryonal tumour of the cerebellum, classically occurring in childhood [1]. Medulloblastoma is exceedingly rare in adults, with an estimated incidence of 0.58 per million per year, and presents as a posterior fossa mass [2]. Clinical presentation in adults can be highly variable and may diverge from the classic headaches, ataxia, and cranial nerve palsies associated with raised intracranial pressure [3]. Posterior fossa tumours can cause audiovestibular symptoms, including hearing loss and vertigo, which can be challenging to differentiate from peripheral vestibulopathies [4]. Diagnosis can be particularly challenging when symptoms are subtle, non-specific, or atypical, as in our case.

Clinical examination can help to distinguish whether vertigo is peripheral or central in nature, determining the subset of patients that should receive neuroimaging. Central vertigo is typically associated with brainstem or cerebellar signs such as gait ataxia, impaired smooth pursuits, and non-fatigable multidirectional nystagmus, whereas peripheral vertigo often yields a positive Dix-Hallpike test and a unidirectional, fatigable nystagmus [5]. In patients presenting with acute vestibular syndrome, the HINTS examination (head impulse, nystagmus, and test of skew) can help delineate central and peripheral lesions [6]. The Unterberger test (also known as the Fukuda stepping test), in which a patient walks on the spot with their eyes closed, can indicate vestibular hypofunction if the patient rotates towards the affected side [7]. Patients with concomitant features of raised intracranial pressure, such as morning headaches, vomiting, and visual disturbance, should also receive prompt neuroimaging [8].

In rare cases, physical examination may be falsely reassuring in patients whose vertigo has a central cause, as the sensitivity and specificity of examination techniques are imperfect. Although the HINTS exam is highly sensitive and specific for central lesions, 3.7% of patients with a reassuring examination ultimately have an underlying central pathology [9].

We describe a case of medulloblastoma in an adult presenting with vertigo and exertional dysacusis (abnormal perception of sound with physical exertion) with an unremarkable neurological examination. We discuss the challenges associated with the diagnosis and management of adults with medulloblastoma, advocating both for early neuroimaging for patients with progressive or atypical vertigo and for the recruitment of similar patients into clinical trials to guide future management.

## Case presentation

A right-handed man in his 30s initially presented to an ENT surgeon with an unusual two-year history of asymmetrical audiovestibular symptoms. After intense exercise such as cycling or lengthy runs, he developed hyperacusis and dysacusis in the left ear. Normal sounds of a certain volume were replaced by a crackling ‘white noise’ sound. Sounds at a quieter volume, such as voice, did not induce the symptoms. The symptoms lasted for a few minutes to a few hours after the stimulus. A magnetic resonance imaging (MRI) brain was requested, which was normal. He reduced the intensity of exercise and so avoided the symptoms.

One year later, he presented to an ENT surgeon with a four-week history of worsening positional vertigo and nausea. This began the morning after a party, and he initially attributed these symptoms to alcohol. He was otherwise fit and well with no significant past medical history.

On examination, he walked with a normal gait. Weber’s test was central, and Rinne’s test was normal bilaterally [10]. His pursuit and saccadic eye movements were normal, as was his head thrust test, and there was no nystagmus, skew, or evidence of cerebellar dysfunction. Romberg’s test, in which the patient stood with their feet together and eyes closed [11], was negative, but on Unterberger’s test, he rotated to the left, consistent with left-sided vestibular hypofunction. A Dix-Hallpike test was negative, and otoscopy was unremarkable bilaterally. Fundoscopy was normal. A provisional diagnosis of acute labyrinthitis was made, and the patient was prescribed a trial of prochlorperazine, given Cawthorne Cooksey exercises, and invited to return after three weeks if no better.

**Figure 1:**
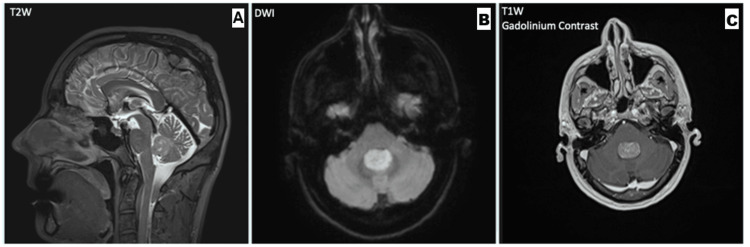
MRI Brain Series (A) Sagittal T2W sequence demonstrating an isointense intraventricular mass without obstructive hydrocephalus. (B) Axial diffusion-weighted MRI demonstrating restricted diffusion (bright). (C) Coronal T1 gadolinium contrast MRI demonstrating homogeneous enhancement. MRI: magnetic resonance imaging; T2W: T2-weighted; DWI: diffusion-weighted imaging; T1W: T1-weighted

He returned to the clinic one month later with additional symptoms of nausea and vomiting, mainly present in the mornings, not associated with vertigo and worse on rolling from his right side onto his back. On examination, gait was still normal, but Unterberger’s test was now positive to the right, where previously it had been positive to the left. His Dix-Hallpike test now provoked disequilibrium but no rotational vertigo, and, in view of the diagnostic uncertainty, he was referred for an MRI brain scan. This demonstrated a well-circumscribed 20 x 13 mm homogeneously enhancing fourth ventricular mass, which was initially thought to represent an ependymoma (Figure 1). There was no hydrocephalus.

Over the next few weeks, the patient developed persistent nausea and vomiting. The severity of the clinical features was out of keeping with the MRI appearances, and so, the MRI was reviewed. It was noted that the diffusion-weighted imaging showed more restricted diffusion than had been previously appreciated, so an alternative diagnosis, such as medulloblastoma, was considered. A whole spine MRI showed no metastatic lesions, and a CT chest, abdomen, and pelvis was unremarkable. Imaging was reviewed in a Neuro-oncology multidisciplinary team meeting, and the patient was scheduled for surgery.

**Figure 2:**
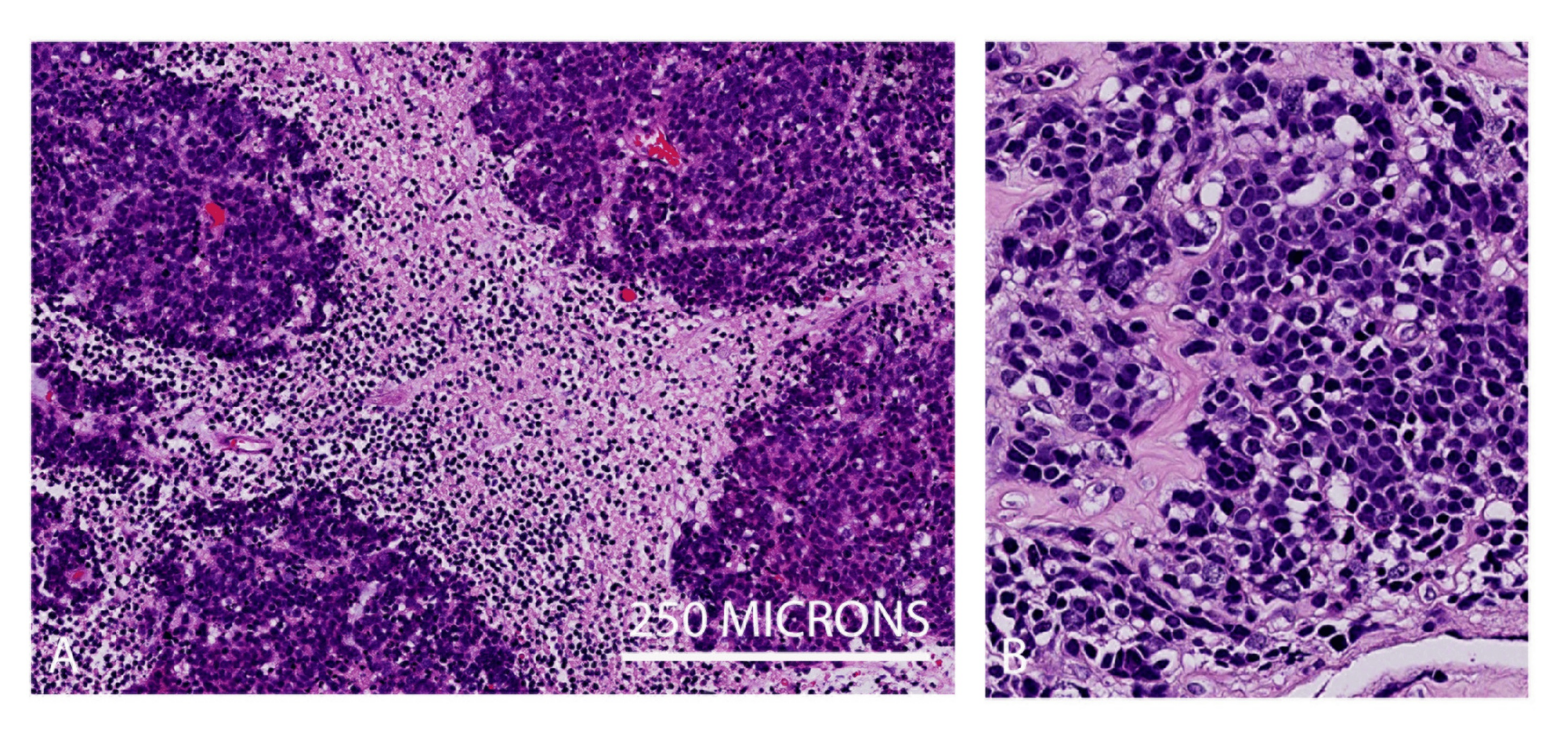
Histology Demonstrating Classic Medulloblastoma (A) H&E shows a highly cellular tumour composed of pleomorphic small cells arranged in sheets and vague lobules but without a significant nodular desmoplastic pattern. (B) Cells at higher magnification show minimal cytoplasm, nuclear moulding but without significant anaplasia. Mitosis and apoptosis were seen.

He then underwent a posterior fossa craniotomy with macroscopic complete resection of the tumour. The final histological diagnosis was classic medulloblastoma CNS WHO Grade 4, and genetic profiling confirmed the non-WNT/non-SHH molecular subgroup (Figure 2). The patient’s nausea and vomiting improved significantly following surgery. He developed diplopia due to a sixth nerve palsy and a complete right facial palsy a week after surgery, which has persisted. A postoperative MRI scan showed no evidence of brainstem ischaemia.

As part of the staging workup, the patient had a lumbar puncture on postoperative day nine, which yielded a cellular sample (WBC 72/mm^3^) of predominantly mononuclear cells and a high CSF protein concentration (2.27 g/L). Cytology demonstrated scattered atypical cells with hyperchromatic nuclei and mitotic figures. Although malignant cells could not be formally identified, the CSF results were felt to be suggestive of metastatic spread within the CNS and, therefore, stage M1 disease.

He received craniospinal irradiation (CSI) to a dose of 39.6 Gy in 22 fractions over 4.5 weeks, followed by a boost of 14.4 Gy in eight fractions to the posterior fossa delivered using RapidArc Intensity Modulated RT (Varian Medical Systems, Palo Alto, CA, US). He then received six weekly Packer chemotherapy (intravenous cisplatin, lomustine, and vincristine).

He remained mildly ataxic with double vision, correctable with prisms, and his MRI brain scan a year after the completion of radiotherapy showed no evidence of recurrence. Recent imaging, two years after the completion of radiotherapy, demonstrated spinal drop metastases, and he has travelled overseas for palliative treatment.

## Discussion

This case illustrates the importance of imaging in patients presenting with atypical and discordant signs of a peripheral vestibulopathy, particularly when expected recovery does not take place. This young patient had satisfactory neurological and HINTS examinations, normal fundoscopy, and a lateralising Unterberger test, all of which suggest vertigo of a peripheral aetiology. However, fourth ventricular tumours may present with isolated vomiting even in the absence of nausea and features of raised intracranial pressure due to obstructive hydrocephalus. However, as in this case, the classic symptoms of gait ataxia, cranial nerve palsies, and raised intracranial pressure may not be present. An unusual aspect of this case was that signs of vestibular hypofunction appeared to move from one side to the other, and there was a longer background of unexplained and unusual audiological symptoms. Re-test asymmetry is a recognised phenomenon in the Unterberger test, hypothesised to be related to emerging central compensation in vestibular disorders [12], though its relevance in this case is unclear.

Posterior fossa tumours, both benign and malignant, can rarely mimic benign positional vertigo, and early neuroimaging should be sought if a space-occupying lesion is suspected. In a recent case series of six patients with seemingly peripheral vertigo with intracranial masses, a 29-year-old female patient with medulloblastoma presented with a three-month history of positional vertigo, without any other symptoms or signs, which resolved after the resection of the tumour [13].

Medulloblastoma, a primitive neuroectodermal tumour derived from granule cell precursors of the cerebellum, was first described in 1925 by Bailey and Cushing [14,15]. The WHO 2021 guidelines now classify medulloblastoma into four categories: WNT-activated, SHH-activated TP53 mutant, SHH-activated TP53 wild type, and non-WNT/non-SHH [16]. Non-WNT/non-SHH tumours with MYC activation are the most aggressive medulloblastoma subclass, with a five-year overall survival (OS) of <60% [17].

While medulloblastoma in children generally carries a favourable prognosis, adults with medulloblastoma have a poorer prognosis, with a five-year OS of around 50%, depending on the extent of resection and the presence of CSF metastases. In our case, it is likely that he had CSF spread at diagnosis, which reduces his five-year survival to about 30%. This is still considerably better than the survival of glioblastoma, the most common malignant primary brain tumour in adults [18].

The existing literature regarding the treatment of adult patients consists of a few single-arm adult studies and single-centre retrospective studies enrolling a low number of patients [19]. Even within the paediatric practice, there is no widely accepted standard of care for patients with high-risk disease. Different strategies have been adopted to improve outcomes in the paediatric population, including the use of high-dose CSI (for example, UK HART, MILAN, HIT 2000, POG 9031, and St Jude MB96/03), high-dose myeloablative chemotherapy with autologous stem cell return (for example, MILAN and French IGR), or concurrent chemotherapy with radiotherapy (COG 99701). However, these intensive paediatric approaches result in significant and unacceptable levels of toxicity (including myelosuppression, ototoxicity, and neurotoxicity) in adult patients. Even with the Packer regimen (lomustine, cisplatin, and vincristine), which is the most used protocol in the adult population, dose modifications may be required [20]. SHH pathway inhibitors, such as sonidegib, are being assessed in clinical trials for adult medulloblastoma, and the European trial EORTC 1634-BTG/NOA-23 is currently evaluating sonidegib as an adjunct to standard chemoradiotherapy in post-pubertal patients with SHH-active disease [21].

Published data have shown that proton beam therapy (PBT) reduces the late toxicities associated with conventional photon CSI [22]. However, proton beam CSI was initially only available for the standard risk medulloblastoma paediatric and teenage and young adult population at the time this patient was treated but is now available in the UK for all standard and high-risk medulloblastoma patients.

Peripheral aetiologies of vertigo are more common than central causes; epidemiological studies estimate that 65% of patients with vertigo have an underlying peripheral cause, while less than 1% are thought to be caused by space-occupying posterior fossa lesions [23]. Clinical examination can theoretically delineate central and peripheral causes of vertigo. Central causes of vertigo are classically associated with brainstem/cerebellar signs including gait and limb ataxia, jerky pursuits, and central non-fatigable vertical/multidirectional nystagmus. In contrast, a positive Dix-Hallpike test approaches 100% sensitivity for peripheral causes of vertigo in patients with less than three risk factors for stroke [24].

The Unterberger test is used clinically to screen for vestibular hypofunction, though some studies have shown no difference in rotation between patients with vestibular disease and controls, and it cannot assess central vertigo [25]. It does not indicate which side is involved, as both left and right pathways can be damaged by any central lesion. There is no good explanation for the disparate findings in the first and second examinations, nor the relationship between the recent vestibular symptoms and the longer history of unusual auditory disturbance, which presented when the MRI scan was normal.

## Conclusions

This unusual case highlights that medulloblastoma should be considered in the differential diagnosis of non-resolving vertigo and vomiting due to a fourth ventricular mass, particularly when the mass shows restricted diffusion on MRI. Given its propensity for metastasis, delayed diagnosis of medulloblastoma increases the likelihood of dissemination within the CNS and poorer outcomes. It is, therefore, important to maintain a high index of suspicion in patients with persistent or progressive symptoms, with prompt neuroimaging in refractory vertigo and vertigo with atypical features. The patient’s treatment was guided by the older paediatric protocols, with less intensive chemotherapy and full-dose CSI, which are no longer used to treat high-risk medulloblastoma in paediatrics. Good-quality clinical trial data, specifically for adults with medulloblastoma, are needed to advance practice and improve outcomes for patients with this rare disease.

## References

[1] Northcott PA, Robinson GW, Kratz CP (2019). Medulloblastoma. Nat Rev Dis Primers.

[2] Giordana MT, Schiffer P, Lanotte M, Girardi P, Chio A (1999). Epidemiology of adult medulloblastoma. Int J Cancer.

[3] Quinlan A, Rizzolo D (2017). Understanding medulloblastoma. JAAPA.

[4] Terakawa Y, Tsuyuguchi N, Takami T, Ohata K (2011). Medulloblastoma manifesting as sudden sensorineural hearing loss. J Korean Neurosurg Soc.

[5] Karatas M (2008). Central vertigo and dizziness: epidemiology, differential diagnosis, and common causes. Neurologist.

[6] Kattah JC, Talkad AV, Wang DZ, Hsieh YH, Newman-Toker DE (2009). HINTS to diagnose stroke in the acute vestibular syndrome: three-step bedside oculomotor examination more sensitive than early MRI diffusion-weighted imaging. Stroke.

[7] Fukuda T (1959). The stepping test: two phases of the labyrinthine reflex. Acta Otolaryngol.

[8] Dunn LT (2002). Raised intracranial pressure. J Neurol Neurosurg Psychiatry.

[9] Ohle R, Montpellier RA, Marchadier V, Wharton A, McIsaac S, Anderson M, Savage D (2020). Can emergency physicians accurately rule out a central cause of vertigo using the HINTS examination? A systematic review and meta-analysis. Acad Emerg Med.

[10] Kelly EA, Li B, Adams ME (2018). Diagnostic accuracy of tuning fork tests for hearing loss: a systematic review. Otolaryngol Head Neck Surg.

[11] Forbes J, Munakomi S, Cronovich HA (2025). Romberg test. StatPearls.

[12] Honaker JA, Shepard NT (2012). Performance of Fukuda Stepping Test as a function of the severity of caloric weakness in chronic dizzy patients. J Am Acad Audiol.

[13] Chen YX, Sun HJ, Mu XT (2022). Intracranial tumors mimicking benign paroxysmal positional vertigo: a case series. Front Neurol.

[14] Bailey P, Cushing H (1925). Medulloblastoma cerebelli: a common type of midcerebellar glioma of childhood. Arch NeurPsych.

[15] Massimino M, Antonelli M, Gandola L (2013). Histological variants of medulloblastoma are the most powerful clinical prognostic indicators. Pediatr Blood Cancer.

[16] Louis DN, Perry A, Wesseling P (2021). The 2021 WHO Classification of Tumors of the Central Nervous System: a summary. Neuro Oncol.

[17] Cho YJ, Tsherniak A, Tamayo P (2011). Integrative genomic analysis of medulloblastoma identifies a molecular subgroup that drives poor clinical outcome. J Clin Oncol.

[18] Kocakaya S, Beier CP, Beier D (2016). Chemotherapy increases long-term survival in patients with adult medulloblastoma--a literature-based meta-analysis. Neuro Oncol.

[19] Padovani L, Sunyach MP, Perol D (2007). Common strategy for adult and pediatric medulloblastoma: a multicenter series of 253 adults. Int J Radiat Oncol Biol Phys.

[20] Luque R, Benavides M, Del Barco S (2021). SEOM clinical guideline for management of adult medulloblastoma (2020). Clin Transl Oncol.

[21] Hau P, Frappaz D, Hovey E (2021). Development of randomized trials in adults with medulloblastoma-the example of EORTC 1634-BTG/NOA-23. Cancers (Basel).

[22] Brown AP, Barney CL, Grosshans DR (2013). Proton beam craniospinal irradiation reduces acute toxicity for adults with medulloblastoma. Int J Radiat Oncol Biol Phys.

[23] Sekine K, Sato G, Takeda N (2005). Incidence of vertigo and dizziness disorders at a university hospital (Article in Japanese). Nihon Jibiinkoka Gakkai Kaiho.

[24] Bodunde O, Regis A, LePage R, Turgeon Z, Ohle R (2019). Does a positive Dix-Hallpike rule out a central cause of vertigo?. CJEM.

[25] Hickey SA, Ford GR, Buckley JG, Fitzgerald O'Connor AF (1990). Unterberger stepping test: a useful indicator of peripheral vestibular dysfunction?. J Laryngol Otol.

